# Stereotactic body radiation therapy as an effective and safe treatment for small hepatocellular carcinoma

**DOI:** 10.1186/s12885-018-4359-9

**Published:** 2018-04-20

**Authors:** Tao Zhang, Jing Sun, Weiping He, Huan Li, Junjie Piao, Huijun Xu, Xuezhang Duan

**Affiliations:** Radiation Oncology Center, Beijing 302 Hospital, Xi Si Huan Middle Road, Fengtai District, Beijing, 100039 China

**Keywords:** Hepatocellular carcinoma, Stereotactic body radiation therapy, CyberKnife, Efficacy, Safety

## Abstract

**Background:**

To evaluate the efficacy and safety of stereotactic body radiation therapy (SBRT) in patients with small hepatocellular carcinoma(sHCC) who were ineligible for surgery or ablation therapies.

**Methods:**

From March 2011 to December 2012, 28 cases with sHCC which were ineligible or refused surgical resection, transplantation or local ablation were treated with CyberKnife SBRT. Median size of tumors was 2.1 cm (range:1.1–3.0 cm), a dose of 10-15Gy per faction was given over 3–6 consecutive days, resulting in a total dose of 35-60Gy.

**Results:**

The median follow-up period was 36 months, with the response rate of complete response (CR) in 17 cases, partial response (PR) in 8 cases, stable disease (SD) in 2 cases and progressive disease (PD) in one case. Overall response rate was 89.28%. Overall survival rates in 1, 2 and 3 years were 92.86, 85.71 and 78.57%, respectively. Local control rates in 1, 2 and 3 years were 96.43, 92.86 and 89.28%, respectively. No grade ≥ 3 hepatic toxicity was observed.

**Conclusion:**

CyberKnife treatment was a safe and effective option for sHCC, which had shown good local control, high overall survival rates and low toxicity. CyberKnife SBRT could be served as an alternative treatment for patients with sHCC which is unsuitable for surgical treatment or local ablation.

## Background

Hepatocelluar carcinoma (HCC) is the most common primary liver tumor and the fifth common malignant disease globally [1]. The annual diagnosis patients are over five hundred thousand [2]. According to the guideline, resection, liver transplantation and radiofrequency ablation(RFA) are the radical treatment options for small hepatocellular carcinoma(sHCC) [3]. However, only 10–30% patients who are diagnosed with sHCC are eligible for curative therapies due to several reasons [4]. Besides, for lack of donor and strict indications, liver transplantation is limited. RFA is the radical treatment option for the patients with sHCC. But if the tumor is close to bile duct or large vessels, or at deep location of the liver, or positioned at the top of the dome etc., the ablative therapy can’t be safely performed. The best treatment of sHCC remains controversial, particularly for sHCC patients who cannot be treated radically. Historically, the conventional radiotherapy for HCC has been limited due to the radiation sensitivity of normal liver, the risk of radiation-induced liver disease (RILD) after the whole liver radiation of low doses, and concern for the adjacent radiation sensitive organs such as stomach, duodenum, etc. [5]. In recent years, the advanced radiation therapy such as stereotactic body radiation therapy (SBRT) has shown high rates of local control for primary and metastatic liver cancers while avoids the risk of RILD. It is the best choice for sHCC patients who are unsuitable for resection, liver transplantation and RFA.

CyberKnife SBRT, which stands for the image guided precision radiation therapy, is the new type of stereotactic radiosurgery. The most important features of CyberKnife treatment are online position correction, respiratory synchronous tracking, real-time tracking and image guided radiotherapy, etc. Respiratory synchronous tracking system guides the accelerator to track the tumor movement, which can control the precision within 1 mm, realizing the precise therapy. Meanwhile, it enhances the dose of tumor region, reduces the dose of normal tissues nearby, and increases the therapeutic gain ratio. The aim of our study is to evaluate the curative effect and security of CyberKnife SBRT in treating sHCC in our hospital.

## Methods

### Patients enrollment

Between March 2011 and December 2012, 28 sHCC patients were treated with CyberKnife SBRT. All patients met the criteria as follows: (a)The max diameter of the tumors ≤3 cm; (b) Without portal vein tumor thrombus and lymphatic metastasis; (c)Without extrahepatic metastasis; (d)unfeasible, difficult or refusing to undergo surgery or percutaneous ablative therapies; (e)Child-Pugh Classification(CPC) A or B; (f) eastern cooperative oncology group(ECOG) score 0 or 1; (g)Single tumor.

The clinical diagnosis was based on the result of imaging according to the international guidelines on the management of HCC [6]. The characteristic tumor appearance was defined by at least two imaging studies (including dynamic enhanced computed tomography (CT) and magnetic resonance imaging (MRI) scans as well as angiograms). All patients were managed in multidisciplinary setting with all legitimate treatment options available and provided with written informed consent before treatment.

Baseline characteristics of 28 patients were listed below (Table 1). Twenty-one men (75.0%) and 7 women (25.0%) were included in the study. The median age was 49 years old (22–65 years old). There were 24 hepatitis B patients (85.7%) and 4 hepatitis C patients (14.3%), in which there were 26 cirrhosis patients (92.86%). Alpha fetoprotein(AFP) level ranged from 1.3 ng/ml to 1000 ng/ml (median 19.3 ng/ml). Median tumor maximum diameter was 2.1 cm (range, 1.1 cm–3.0 cm). Ten patients (35.7%) had no previous treatment records and 18 patients (64.3%) were previously treated with procedures such as transcatheter arterial chemoembolization(TACE), RFA, etc. Among the 18 patients who had treatment history, 7 of them suffered recurrence within the liver outside the treated lesion, while the other 11 had incomplete responses after TACE, which were diagnosed by a characteristic tumor appearance by two imaging studies (including dynamic enhanced CT and MRI scans). Seasoned imaging doctors have provided assistance in diagnosis. Before enrollment, 19 hepatitis B patients had received antiviral treatment with entecavir or adefovir, and the serum HBV-DNA levels were all below 40 IU/ml. Another 5 hepatitis B patients, whose serum HBV-DNA levels showed positive, received antiviral treatment with entecavir after the enrollment. The 4 hepatitis C patients had also received antiviral treatment with peg-interferon and ribavirin before enrollment, and the serum HCV-RNA levels were all below 15 IU/ml.

**Table 1:** Clinical and biochemical characteristics of participants enrolled in the study

Variables	*n* (%)
Sex	
Male	21 (75.0)
Female	7 (25.0)
Age (years)	
<60	20 (71.4)
≥60	8 (28.6)
Type of chronic hepatitis	
Hepatitis B	24 (85.7)
Hepatitis C	4 (14.3)
Cirrhosis	
Yes	26 (92.9)
No	2 (7.1)
AFP(ng/ml)	
<200	22 (78.6)
≥200	6 (21.4)
Child-Pugh classification	
A	24 (85.7)
B	4 (14.3)
ECOG Score	
0	25 (89.3)
1	3 (10.7)
Tumor diameter (cm)	
≤2 cm	14 (50.0)
>2 cm,≤3 cm	14 (50.0)
Use of fiducials in SBRT	
Yes	27 (96.4)
No	1 (3.6)
Previous treatments	
No	10 (35.7)
Resection	2 (7.1)
TACE	13 (46.4)
Resection+TACE	1 (3.6)
TACE+RFA	1 (3.6)
TACE+PCA	1 (3.6)

NOTE. Data presented as No (%)

*Abbreviations:* AFP alpha-fetoprotein, *SBRT* stereotactic body radiation therapy, *TACE* transarterial chemoembolization, *RFA* radiofrequency ablation, *PCA* percutaneous cryoablation

### Cyberknife therapy

Fiducial implantation: 27 patients were implanted 3 to 5 fiducials 7 days prior to receiving CyberKnife treatment. The fiducials were 6 cm from tumors in the vicinity. The implantation of fiducials was to provide target tracking during CyberKnife treatment. Twenty seven patients with the implanted fiducials adopted respiratory tracking and fiducial tracking techniques simultaneously, while one patient adopted X-sight spine tracking techniques.

CT localization: CT scan was undergone 1 week after fiducial implantation. In addition to benchmark image as plain CT scans, the auxiliary images were chosen based on patients’ conditions, and these images are enhanced CT scans, MRI, positron emission tomography-computed tomography(PET-CT) and hepatic arteriography, etc. The acquired parameters of CT images are as follows: tilted angel of 0^o^; slice thickness of 1 mm; voltage of 120 KV tube current of 400 mA; scan length of 20 cm; pixel size of 512 × 512. The scan is head first with dorsal decubitus.

CyberKnife treatment plan design: Radiation therapists delineated the gross target volume (GTV) and organs at risk (normal liver, stomach, bowel, duodenum, kidneys and spine cord). Planning target volume (PTV) expands 3-5 mm of GTV. All plans were designed by G4 CyberKnife MultiPlan (Version 4.0.2). A total dose of 35-60Gy was delivered in 3–6 fractions. The treatment plan enclosed PTV with 72–80% isodose line of maximum dose quated to the prescribed dose. In addition, normal tissues dose was within normal radiotherapy tolerance dose (TG-101 report). CyberKnife treatment plan was transferred into treatment control system. The treatment will be started after data verification.

### Post-therapy evaluation and follow-up

Follow-up period was defined from the last treatment to patient’s loss or death. Follow-up was done every 3 months in the first year, then 3 or 6 months thereafter. A review of each patient’s prior medical history, physical examination, complete blood counts, biochemical profiles, tumor markers, liver function and imaging examinations such as enhanced abdominal CT scans or MRI were performed at each follow-up.

Short-term therapeutic evaluation was based on Modified Response Evaluation Criteria in Solid Tumors (mRECIST) [7]. Complete response (CR) means all target tumor disappear in the enhanced CT images of arterial phase; partial response (PR) means the diameter sum of the target tumor reduces more than 30% in the arterial phase enhancement CT images; progressive disease (PD) means the diameter sum of the target tumor increases more than 20% in contrast CT images on the arterial phase, or new tumor appears; stable disease (SD) means reduced or increased volume between PR and PD. Long-term therapeutic evaluation depended on overall survival (OS) and local control (LC) at 1-year, 2-year and 3-year, and LC rate indicates the proportion of the patients with no tumor lesion pregress. Toxicity reaction evaluation was on the basis of the Common Terminology Criteria for Adverse Events (CTCAE) version 4.0.J AM ACAD Dermatol [8].

### Statistical analysis

SPSS 19.0 was used for statistical analysis. A *P* value < 0.05 was defined as the threshold of statistical significance. The overall survival and local control rates were estimated using the Kaplan-Meier method. Factors significantly associated with OS and LC were identified by multivariate analysis using a stepwise Cox model, with calculation of hazard ratios (HRs) and 95% confidence intervals (CIs).

## Results

### Short-term effect

All 28 patients completed the treatments. After 3–6 months of SBRT, there were 17 patients with CR (60.71%), 8 patients with PR (28.57%), 2 patients with SD (7.14%) and 1 patient with PD (3.58%). The response rate was (CR + PR)/28 × 100% = 89.28%, and the disease control rate was (CR + PR + SD)/ 28 × 100% = 96.42%.

### Long-term effect

**Fig. 1:**
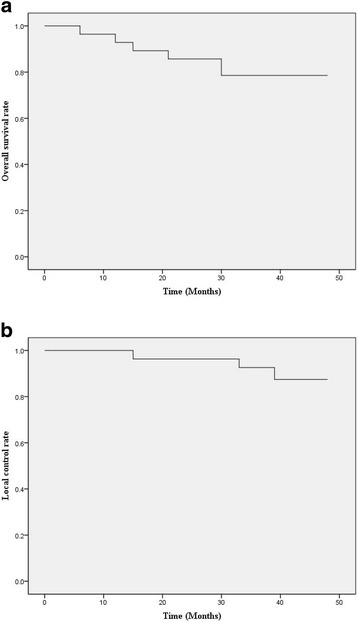
Overall survival and local control rates. **a** The overall survival rates of 1-year, 2-year and 3-year were 92.86, 85.71 and 78.57%, respectively. **b** The local control rates of 1-year, 2-year and 3-year were 96.43, 92.86 and 89.28%, respectively

**Fig. 2:**
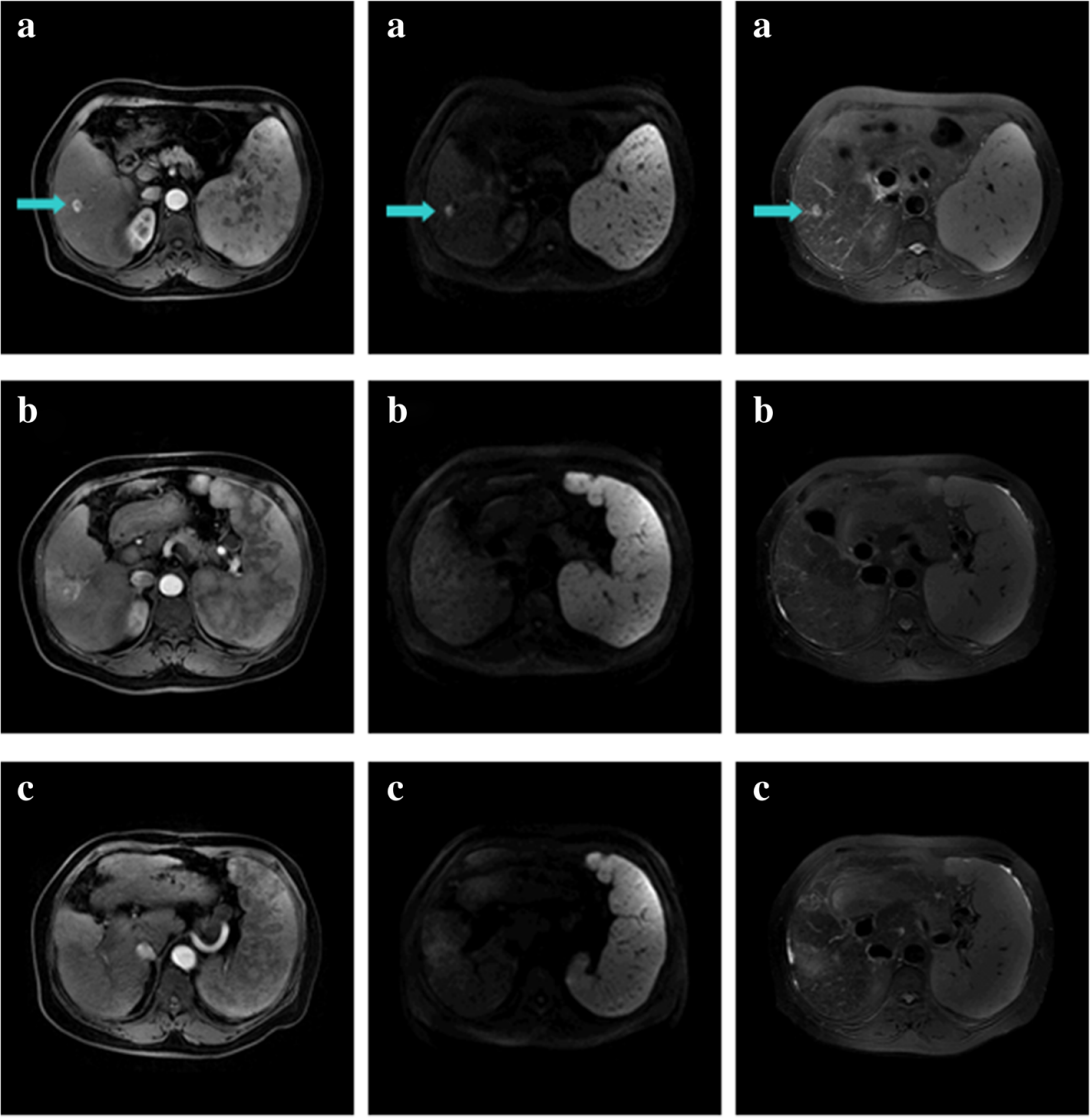
Example of complete response to SBRT assessed by MRI. **a** The initial abdominal MRI scan with the primary HCC indicated by the arrow. **b** MRI scan of 6 months after SBRT. This patient was classified as CR at 6 months after SBRT. **c** MRI scan of 3 years after SBRT. The lesion in liver reveals changes after treatment and no activity

The median follow-up was 36 months (3-53 months). By October 2015, among the enrolled 28 patients, 6 patients died, in which 2 patients died of upper gastrointestinal bleeding, 1 hepatic encephalopathy, 1 hepatorenal syndrome, 1 hepatic failure and 1 obstruction of biliary tract. The overall survival rates of 1-year, 2-year and 3-year were 92.86, 85.71 and 78.57%, respectively. The local control rates of 1-year, 2-year and 3-year were 96.43, 92.86 and 89.28%, respectively (Fig. 1). An example of complete response to SBRT assessed by MRI was showed below (Fig. 2).

### Adverse events

All scheduled treatments were completed without manifestations of toxicity. Acute toxicity reaction including abdominal pain, fatigue, vomiting and anorexia could be relieved gradually by symptomatic treatment. One patient experienced grade 4 hyperbilirubinemia and died of liver failure. However, the patient was classified as CPC-A before the radiotherapy and the tumor diameter was only within 3 cm, so it is hard to distinguish liver failure caused by radiotherapy from that caused by other factors. Grade 1 aminopherase elevation was observed in 6 patients, and none of the patients have ALP elevation. After the symptomatic treatment, the laboratory test results returned to normal. The rest patients were with steady liver function during and after the therapy. Grade 1–2 hematotoxicity was manifested as thrombocytopenia and aleucocytosis. And Recombinanthuman granulocyte-colony stimulating factor (rhG-CSF) was prescribed to treat aleucocytosis. However, conservative method was applied to thrombocytopenia, and the platelet elevated gradually after the radiotherapy. None severe gastrointestinal complication happened, such as haemorrhage and perforation.

### Clinical factors influencing OS and LC

To investigate the risk factors for overall survival rate and local control rate, clinical data, including sex, age, type of chronic hepatitis, cirrhosis, AFP, CPC and any other characteristics were analyzed. The results were all with no statistical significance (all *P*>0.05; Table 2). We also compared the overall survival rate, local control rate of two groups of patients, among which one group had previous treatment history while the other didn’t. The results showed no statistical significance (OS *P* = 0.774, LC *P* = 0.415) as well.

**Table 2:** Multivariate analysis of risk factors for overall survival and local control

Factor	Overall survival			Local control		
	HR	95%CI	*P*	HR	95%CI	*P*
Sex	0.689	0.061–7.771	0.763	0.725	0.160–3.296	0.678
Age	0.764	0.078–7.496	0.817	1.348	0.274–6.623	0.713
Type of chronic hepatitis	0.941	0.096–9.189	0.958	0.583	0.097–3.505	0.555
Cirrhosis	0.075	0.004–1.275	0.073	0.767	0.054–10.915	0.845
AFP	0.546	0.132–2.263	0.404	0.921	0.221–3.845	0.910
Child	0.080	0.003–2.250	0.138	0.636	0.067–6.053	0.694
ECOG	0.786	0.108–5.728	0.812	3.011	0.439–20.627	0.262
Tumor diameter (cm)	3.395	0.724–15.926	0.121	1.218	0.308–4.825	0.778
Previous treatments	1.209	0.404–3.620	0.734	0.796	0.301–2.104	0.645

### Patients status after CyberKnife therapy

Thirteen patients were with tumor relapsed, among which 3 patients were infield, 9 patients were outfield, 1 patient was with metastasis to lung. The median recurrence time was 23.5 months (range, 6–52 months) after radiotherapy. Among the recurrent patients, 9 patients received TACE, 1 patient adopted RFA, 3 patients were retreated with CyberKnife SBRT.

## Discussion

For patients with single lesion, especially sHCC, resection is the most effective treatment, with which the 5-year overall survival rate may run up to 60–70% [9, 10]. Sadly, merely a small part of the patients could receive the resection owing to the discompensated liver function, abnormal function of blood coagulation and the tumor positioning in the deep section of the liver, etc. [11]. Most patients cannot benefit from liver transplantation for the lack of donor and high expenses. For patients who are unsuitable for resection, percutaneous ablation therapy (RFA, anhydrous alcohol injection) are the radical procedures. The 5-year overall survival rate of sHCC patients with CPC-A who received RFA is same with the resection [12]. However, for tumors close to gallbladder, bile duct or large vessels, or near the diaphragm which is at the top of liver, the treatment is limited [13]. With the advancement of the radiation therapy, radiotherapy becomes one of the best choices for sHCC patients unsuitable for resection or RFA.

CyberKnife SBRT, featured with accurate high-dose radiotherapy, was first applied to recurrent squamous cell carcinoma of head and neck patients who have no resection options or not suitable for surgery [14]. Blomgren first applied SBRT to liver cancer patients with primary tumor or the secondary tumor who were unsuitable for surgery, TACE and RFA 20 years ago [15]. The results showed higher local control rate and lower toxicity reaction rate [16]. At present, HCC SBRT is the remedy radiotherapy for incomplete embolization of postoperative patients who received TACE, transitional therapy of preoperative transplantation of liver and the option of recurrence HCC [17, 18].

Yoon SM and his coworkers reported 93 sHCC patients treated with SBRT, whose 1-year and 3-year OS was 86 and 53.8%, respectively, tumor diameter less than 6 cm, tumor number less than three, single fraction dose 10-20Gy, total doses 30-60Gy. They considered SBRT could be served as an alternative option for the patients unsuitable for other radical treatments [19]. In our research, the 1-year OS and 3-year OS were 92.86 and 78.57%, which were higher than the results above. We consider that with single dose of 10-15Gy and total doses of 35-60Gy, SBRT is an optional choice to treat sHCC patients whose diameter of tumor is less than 3 cm.Yuan Z retrospectively analyzed and compared the treatment effect of the CyberKnife SBRT and surgical resection of Barcelona stage I HCC patients. The result showed that the 1-year, 2-year and 3-year overall survival rates of the CyberKnife SBRT were 72.7, 66.7 and 57.1%, respectively, while the surgical resection were 88.5, 73.1 and 69.2% respectively. Numerically, the OS of resection was higher than radiotherapy. However, there were no statistical differences between them. The study showed equivalent curative effect between CyberKnife treatment and resection for the early-stage HCC patients [20]. Sylvain Dewas reviewed 120 patients who received CyberKnife treatment, among which 42 were HCC patients, 72 metastatic liver cancer, 6 cholangicellular carcinoma. The median diameter was 33 mm, median GTV was 32.38cm^3^, and median total doses were 45Gy. The result indicated local control rates at 1-year and 2-year were 84 and 74.6%. In the meanwhile, total doses, tumor diameter and tumor volume were considered as the prognostic factors [21]. We analyzed clinical characteristics including sex, age, type of chronic hepatitis, etc., to discuss factors that may affect overall survival and local control rates. The analyzed results showed no statistical significance (Table 2). We supposed that this was caused by the small size of studied patients. We have conducted further studies by enrolling more patients to prove our conclusions.

Grade I and II hematotoxicity and hepatotoxicity were the main toxicity in our study, which was characterized with low-grade aminopherase elevation, bilirubin elevation, thrombocytopenia and aleucocytosis, and most of them could return to normal gradually after radiotherapy. Only one patient with hepatitis B liver cirrhosis suffered liver failure after 3 months of radiotherapy, who did not have liver enlargement, massive ascites and viral replication. For liver cirrhosis patients, the causes of liver function damage were complex and hard to figure out. Whereas for patients whose tumor was controlled well after radiotherapy and liver function failure wasn’t caused by tumor progress, they were more likely to suffer from serious liver injury with RILD. RILD was the major obstacles of HCC radiotherapy, and the incidence rate of liver radiotherapy was 5–10% when the dose of whole liver achieved 30-35Gy. The main inducing factors of RILD are the basic liver function, the liver cirrhosis and the PTV range [22]. Jung J predicted the RILD risk of SBRT on sHCC which cannot be resected according to dose-volume parameters, by which he discovered the risk for the cirrhosis patients with CPC-B suffering grade II hepatotoxicity was higher [23]. To evaluate liver function, indocyanine green test which is widely used in preoperation is more sensitive than CPC. However, CPC is much more important than indocyanine green test to forecast the risk of RILD [24].

## Conclusion

CyberKnife SBRT is proved to be effective, with its main advantages being synchronous respiratory tracking, high dose delivery and low fraction [25]. Moreover, the treatment period is shorter. From the result of our study, CyberKnife SBRT was shown to be an effective and safety therapy for sHCC, which achieved higher local control rates, overall survival rates and lower toxicity reaction. The higher dose brought better local control rates but causing the risk of RILD. The optimal dose and hypofraction scheme are still controversial. Moreover, longer follow-up is required to evaluate the correlation between dose-response and potential late toxicity. SBRT achieved same effect with surgical operation. Our study had shown that nine of thirteen recurrent patients were outfield recurrence, which was the main cause of recurrence. And CyberKnife SBRT may improve curative effect when combined with chemotherapy or targeted therapy.

## Abbreviations

AFP: Alpha fetoprotein
CIs: Confidence intervals
CPC: Child-Pugh classification
CR: Complete response
CT: computed tomography
CTCAE: Common Terminology Criteria for Adverse Events
ECOG: Eastern cooperative oncology group
GTV: Gross target volume
HCC: Hepatocellular carcinoma
HRs: Hazard ratios
LC: Local control
mRECIST: Modified Response Evaluation Criteria in Solid Tumors
MRI: Magnetic resonance imaging
OS: Overall survival
P: *P*-value
PD: Progressive disease
PET-CT: Positron emission tomography-computed tomography
PR: Partial response
PTV: Planning target volume
RFA: Radiofrequency ablation
rhG-CSF: Recombin- anthuman granulocyte-colony stimulating factor
RILD: Radiation-induced liver disease
SBRT: Stereotactic body radiation therapy
SD: Stable disease
TACE: Transcatheter arterial chemoembolization

## References

[1] Torre LA, Bray F, Siegel RL, Ferlay J, Lortet-Tieulent J, Jemal A (2015). Global cancer statistics, 2012. CA Cancer J Clin.

[2] Chen W, Zheng R, Baade PD, Zhang S, Zeng H, Bray F (2016). Cancer statistics in China, 2015. CA Cancer J Clin.

[3] Bruix J, Sherman M (2011). American Association for the Study of liver D. Management of hepatocellular carcinoma: an update. Hepatology.

[4] Lau WY, Lai EC (2007). Salvage surgery following downstaging of unresectable hepatocellular carcinoma--a strategy to increase resectability. Ann Surg Oncol.

[5] Lawrence TS, Robertson JM, Anscher MS, Jirtle RL, Ensminger WD, Fajardo LF (1995). Hepatic toxicity resulting from cancer treatment. Int J Radiat Oncol Biol Phys.

[6] European Association For The Study Of The L, European Organisation For R, Treatment Of C (2012). EASL-EORTC clinical practice guidelines: management of hepatocellular carcinoma. J Hepatol.

[7] Lencioni R, Llovet JM (2010). Modified RECIST (mRECIST) assessment for hepatocellular carcinoma. Semin Liver Dis.

[8] Chen AP, Setser A, Anadkat MJ, Cotliar J, Olsen EA, Garden BC (2012). Grading dermatologic adverse events of cancer treatments: the common terminology criteria for adverse events version 4.0. J Am Acad Dermatol.

[9] Bruix J, Boix L, Sala M, Llovet JM (2004). Focus on hepatocellular carcinoma. Cancer Cell.

[10] Bruix J, Sherman M (2005). Practice guidelines committee AAftSoLD. Management of hepatocellular carcinoma. Hepatology.

[11] Rahbari NN, Mehrabi A, Mollberg NM, Muller SA, Koch M, Buchler MW (2011). Hepatocellular carcinoma: current management and perspectives for the future. Ann Surg.

[12] Lencioni R (2010). Loco-regional treatment of hepatocellular carcinoma. Hepatology.

[13] Decadt B, Siriwardena AK (2004). Radiofrequency ablation of liver tumours: systematic review. Lancet Oncol.

[14] Comet B, Kramar A, Faivre-Pierret M, Dewas S, Coche-Dequeant B, Degardin M (2012). Salvage stereotactic reirradiation with or without cetuximab for locally recurrent head-and-neck cancer: a feasibility study. Int J Radiat Oncol Biol Phys.

[15] Blomgren H, Lax I, Naslund I, Svanstrom R (1995). Stereotactic high dose fraction radiation therapy of extracranial tumors using an accelerator. Clinical experience of the first thirty-one patients. Acta Oncol.

[16] Klein J, Dawson LA (2013). Hepatocellular carcinoma radiation therapy: review of evidence and future opportunities. Int J Radiat Oncol Biol Phys.

[17] Murray LJ, Dawson LA (2017). Advances in stereotactic body radiation therapy for hepatocellular carcinoma. Semin Radiat Oncol.

[18] Gkika E, Schultheiss M, Bettinger D, Maruschke L, Neeff HP, Schulenburg M (2017). Excellent local control and tolerance profile after stereotactic body radiotherapy of advanced hepatocellular carcinoma. Radiat Oncol.

[19] Yoon SM, Lim YS, Park MJ, Kim SY, Cho B, Shim JH (2013). Stereotactic body radiation therapy as an alternative treatment for small hepatocellular carcinoma. PLoS One.

[20] Yuan Z, Tian L, Wang P (2013). Comparative research on the efficacy of cyberknife and surgical excision for stage Ihepatocellular carcinoma. Onco Targets Ther.

[21] Dewas S, Bibault JE, Mirabel X, Fumagalli I, Kramar A, Jarraya H (2012). Prognostic factors affecting local control of hepatic tumors treated by stereotactic body radiation therapy. Radiat Oncol.

[22] Cheng JC, Wu JK, Huang CM, Liu HS, Huang DY, Cheng SH (2002). Radiation-induced liver disease after three-dimensional conformal radiotherapy for patients with hepatocellular carcinoma: dosimetric analysis and implication. Int J Radiat Oncol Biol Phys.

[23] Jung J, Yoon SM, Kim SY, Cho B, Park JH, Kim SS (2013). Radiation-induced liver disease after stereotactic body radiotherapy for small hepatocellular carcinoma: clinical and dose-volumetric parameters. Radiat Oncol.

[24] Lee IJ, Seong J, Shim SJ, Han KH (2009). Radiotherapeutic parameters predictive of liver complications induced by liver tumor radiotherapy. Int J Radiat Oncol Biol Phys.

[25] Martin A, Gaya A (2010). Stereotactic body radiotherapy: a review. Clin Oncol.

